# Percutaneous transcatheter closure of high-risk patent foramen ovale in the elderly

**DOI:** 10.1007/s00380-019-01379-0

**Published:** 2019-03-13

**Authors:** Hiroya Takafuji, Shinobu Hosokawa, Riyo Ogura, Yoshikazu Hiasa

**Affiliations:** Department of Cardiology, Tokushima Red Cross Hospital, 103 Irinokuchi, Komatsushima-cho, Komatsushima, Tokushima 773-8502 Japan

**Keywords:** Patent foramen ovale, Percutaneous transcatheter closure, Elderly

## Abstract

The efficacy of percutaneous transcatheter closure for preventing recurrent cerebrovascular events in elderly patients with high-risk patent foramen ovale (PFO) remains unclear, whereas in young patients, it has been shown to effectively prevent the recurrence of embolic stroke. The aim of this study was to investigate the safety and efficacy of percutaneous PFO closure in elderly patients with high-risk PFO. Between September 2012 and October 2018, 14 patients ≥ 60 years old with high-risk PFO underwent percutaneous closure to prevent recurrence of cerebrovascular events. The primary end point was recurrence of cerebrovascular events after closure in elderly patients with high-risk PFO, and the secondary end points were occurrence of device-related complications, cerebral hemorrhage, and new-onset atrial fibrillation (AF). The mean patient age and number of cerebrovascular events before closure were 75.2 ± 6.5 years and 1.7 ± 0.7, respectively. All procedures were successfully performed under general anesthesia by transesophageal echocardiography and using a 25-mm Amplatzer Cribriform device. No procedure-related complications occurred. Patients were followed up for a mean 2.6 ± 1.8 years. No patients experienced device-related complications or recurrent cerebrovascular events. However, one patient had AF-related device closure complications at 1 month postoperatively. In addition, other patient had a cerebral hemorrhage with unknown relationship to PFO closure 3 years postoperatively. Percutaneous closure of high-risk PFO in elderly patients may be as effective and safe as in younger patients. It is crucial to evaluate PFO morphology regardless of age in cases of paradoxical embolism.

## Introduction

Percutaneous transcatheter closure of the patent foramen ovale (PFO) to prevent recurrence of paradoxical embolic stroke has been established as a safe and effective practice [1–3]. However, most studies did not include elderly patients (age ≥ 60 years), and little is known about the clinical benefit of percutaneous PFO closure in older patients. In addition, previous studies have shown that recurrent cerebral ischemia after PFO closure was higher in older than in younger patients [4, 5]. However, PFO-related recurrent cerebral ischemia was not sufficiently evaluated in these studies. Elderly patients generally have other causes of stroke risk such as atherosclerosis, cardiac arrhythmia, and small artery (lacunar) disease. Device closure for PFO is only effective for preventing PFO-related paradoxical embolic stroke. On the other hand, previous studies have demonstrated that PFO morphology was associated with higher stroke recurrence [6]. Furthermore, the Device Closure Versus Medical Therapy for Cryptogenic Stroke Patients With High-risk Patent Foramen Ovale (DEFENSE-PFO) trial confirmed that high-risk PFO closure, as defined by transesophageal echocardiography (TEE) findings, significantly lowered the stroke recurrence rate compared with medical therapy alone [7]. Therefore, PFO closure in elderly patients may be effective in preventing the recurrence of high-risk PFO-related cerebrovascular events. The present study aimed to investigate the efficacy and safety of percutaneous transcatheter PFO closure in preventing recurrence of cerebrovascular events in patients with high-risk PFO over 60 years old.

## Methods

### Study design

This was a single center, retrospective cohort study. The patients who underwent percutaneous PFO closure to prevent recurrence of cerebrovascular events in the Tokushima Red Cross Hospital, Japan, between September 2012 and October 2018 were enrolled.

### Indication of percutaneous PFO closure and definition of high-risk PFO

Cerebrovascular events such as transient ischemic attack (TIA) and PFO-related stroke were diagnosed by a neurologist. TIA was defined as a brief neurological dysfunction. Stroke type was classified according to the embolic strokes of undetermined source (ESUS) criteria [8]. ESUS was defined as an episode with no major cause such as large artery atherosclerotic stenosis, small artery (lacunar) disease, major risk source of cardioembolism, and unusual causes (e.g., arteritis, dissection, vasospasm). Among them, PFO-related ESUS (i.e., paradoxical embolic stroke) was defined as an episode without any other possible causes of embolic stroke (e.g., significant aortic arch atherosclerotic plaques, atrial arrhythmia, atrial appendage thrombus, appendage stasis with reduced flow velocities, and low ejection fraction) but PFO.

PFO was diagnosed by TEE with agitated saline bubble testing. The presence of PFO was defined as a shunt bubble grade > 1. Shunt bubble grade was classified according to the number of passing bubbles from the right to the left atrium: grade 0, none; grade 1, 1–5 bubbles; grade 2, 6–20 bubbles; grade 3, ≥ 20 bubbles [9]. We defined high-risk PFO as follows: (1) long tunnel ≥ 10 mm and PFO separation height ≥ 2 mm, (2) PFO with the presence of hypermobile atrial septal aneurysm (ASA), (3) spontaneous right-to-left shunt through the PFO at rest, and (4) grade 3 shunt bubble through the PFO [7, 10–14]. If these TEE findings were confirmed, the brain–heart team consisting of an interventional cardiologist and neurologist discussed whether to perform percutaneous closure or medical follow-up, also taking into account each patient's frailty and activities of daily living performance. All procedures were performed in accordance with institutional ethical guidelines after informed consent was given by the patients.

### Study design

From September 2012 to October 2018, a total of 14 patients older than 60 years with high-risk PFO-associated cerebrovascular events underwent percutaneous PFO closure in our institution. The primary end point of this study was the recurrence rate of cerebrovascular events (TIA and paradoxical embolic stroke), and the secondary end points were occurrence of device-related complications, cerebral hemorrhage, and new-onset atrial fibrillation (AF) through the study period.

### Percutaneous PFO closure procedure

Percutaneous closure was performed under general anesthesia in a hybrid operating room. The procedure was performed by experienced interventional cardiologists. All cases were performed using TEE guidance. Device size was decided according to balloon size. The used device was the Amplatzer Cribriform (Abbott, Chicago, Illinois).

### Clinical assessments

Clinical assessments were regularly scheduled at 1, 3, 6, and 12 months after the procedure and annually thereafter. Transthoracic echocardiography (TTE) was performed to assess device-related complications: device embolization, device thrombosis, erosion, and residual shunting. All data were retrospectively obtained by medical records or telephone interview with the patient or with family members to determine the occurrence of complications, hemorrhage, and new-onset AF throughout the study period.

### Medications after percutaneous PFO closure

After percutaneous PFO closure, medications were prescribed according to the interventional cardiologist and neurologist’s judgment. Antiplatelet and/or anticoagulants were continued by each patient and chosen on the basis of individual risk-to-benefit ratio. The duration of medical therapy was decided individually for each patient.

### Statistical analysis

All analyses were performed using JMP version 8 (SAS Institute, Cary, NC, USA). Continuous variables are expressed as mean ± SD.

## Results

### Patient, PFO, and procedure characteristics

Baseline patient characteristics are summarized in Table 1. The mean age was 75.2 ± 6.5 years, and 57.1% were men. The prevalence of hypertension, diabetes mellitus, and dyslipidemia was 64.3%, 21.4%, and 57.1%, respectively. Deep vein thrombus was noted in two patients (14.3%). The indication for PFO closure was stroke in most cases. Half of the patients with stroke had multiple episodes. The average number of cerebrovascular events before percutaneous PFO closure was 1.7 ± 0.7 times (range, 1–3 times). TEE findings are shown in Table 2. The mean PFO length and height were 3.2 ± 0.9 and 3.9 ± 1.9 mm, respectively. Hypermobile ASA was observed in 57.1% of patients. A right-to-left shunt through the PFO at rest was detected in ten patients. A grade 3 shunt was observed in 85.7% of patients. Procedural characteristics are shown in Table 3. Procedural success was achieved in all cases. None of major complications occurred during the procedures.

**Table 1:** Baseline patient characteristics

	*n *= 14
Clinical characteristics	
Age, years	75.2 ± 6.5
60–69 years (%)	4 (28.6)
70–79 years (%)	6 (42.8)
80–89 years (%)	4 (28.6)
Male (%)	8 (57.1)
Body mass index, kg/m^2^	22.3 ± 3.4
Hypertension (%)	9 (64.3)
Diabetes mellitus (%)	3 (21.4)
Dyslipidemia (%)	8 (57.1)
Chronic kidney disease (%)	5 (35.7)
Current smoker (%)	3 (21.4)
Migraine (%)	0 (0)
AF (%)	0 (0)
Deep vein thrombus (%)	2 (14.3)
Coronary artery disease (%)	1 (7.1)
Type of cerebrovascular event	
Stroke (%)	12 (85.7)
Multiple infarction (%)	6 (42.8)
TIA (%)	2 (14.3)
Number of cerebrovascular events, times (range)	1.7 ± 0.7 (1–3)

Values are *n* (%) or mean ± SD

*AF* atrial fibrillation, *TIA* transient ischemic attack

**Table 2:** Transesophageal echocardiography findings

	*n *= 14
PFO length, mm	3.2 ± 0.9
Long tunnel ≥ 10 mm (%)	4 (28.6)
PFO height, mm	3.9 ± 1.9
Height ≥ 2 mm (%)	12 (85.7)
Hypermobile ASA (%)	8 (57.1)
Right-to-left shunt at rest (%)	10 (71.4)
Shunt grade 3 with bubble test (%)	12 (85.7)

Values are *n* (%) or mean ± SD

*PFO* patent foramen ovale, *ASA* atrial septal aneurysm

**Table 3:** Procedure characteristics

	*n *= 14
General anesthesia (%)	14 (100)
Procedure time, min	46.0 ± 11.5
TEE guide (%)	14 (100)
Device distribution	
Amplatzer Cribriform (%)	14 (100)
Balloon size diameter, mm	7.7 ± 2.8
Device size	
25 mm	14 (100)
Procedure success (%)	14 (100)
Major complications (%)	0 (0)
Hospital stay after procedure, days	4.4 ± 1.2

Values are *n* (%) or mean ± SD

*TEE* transesophageal echocardiography

### Follow-up and outcomes

Changes in medication after PFO closure are listed in Table 4. Approximately 71% of patients were prescribed single antiplatelet plus anticoagulant therapy at discharge. At 1 month, 35.7% of patients were prescribed single antiplatelet or anticoagulant therapy. Further, most patients were prescribed a single antiplatelet agent or no medications at 6 months, a trend which continued over 2 years.

**Table 4:** Medications after percutaneous patent foramen ovale closure

	Discharge *n *= 14	1 month *n *= 14	6 months *n *= 11	1 year *n *= 10	2 year *n *= 9
Dual antiplatelet therapy (%)	3 (21.4)	0 (0)	0 (0)	0 (0)	0 (0)
Dual antiplatelet therapy and anticoagulant therapy (%)	0 (0)	0 (0)	0 (0)	0 (0)	0 (0)
Single antiplatelet therapy and anticoagulant therapy (%)	10 (71.4)	9 (64.3)	1 (9.1)	1 (10.0)	1 (11.1)
Single antiplatelet therapy (%)	1 (7.1)	4 (28.6)	5 (45.5)	4 (40.0)	4 (44.4)
Anticoagulant therapy (%)	0 (0)	1 (7.1)	1 (9.1)	1 (10.0)	1 (11.1)
No antiplatelet and anticoagulant therapy (%)	0 (0)	0 (0)	4 (36.3)	4 (40.0)	3 (33.3)

Values are *n* (%)

Table 5 presents clinical outcomes after percutaneous PFO closure. During the follow-up period (mean, 2.6 ± 1.8 years), no patients experienced recurrent cerebrovascular events. In addition, no patients had device-related complications, and significant residual device shunt was not observed during the follow-up period. One patient on single antiplatelet therapy suffered cerebellar hemorrhage at 3 year after procedure. New-onset AF was detected in one patient at 1 month postoperatively. However, there were no deaths during the follow-up period.

**Table 5:** Clinical outcomes after patent foramen ovale closure

	*n *= 14
Paradoxical embolism of stroke (%)	0 (0)
TIA (%)	0 (0)
Hemorrhagic stroke (%)	1 (7.1)
Device-related complications (%)	0 (0)
New-onset Af (%)	1 (7.1)
Survival (%)	14 (100)

Values are *n* (%)

*TIA* transient ischemic attack, *Af* atrial fibrillation

## Discussion

In this study, we found no recurrence of cerebrovascular events or any significant complications after high-risk PFO closure in our sample of elderly subjects.

The presence of PFO is associated with paradoxical embolic stroke in both young and elderly subjects [15–17]. However, the existing evidence of percutaneous PFO closure efficacy in preventing paradoxical embolic stroke in elderly subjects is conflicting, and no randomized trials are currently available. Previous studies showed paradoxical embolic stroke can be prevented in both young and elderly patients. Kiblawi et al. reported there was no significant difference in the rate of recurrent stroke/TIA after percutaneous PFO closure regardless of age [18]. Spies et al. reported the incidence of recurrent cryptogenic thromboembolic events after percutaneous PFO closure was not significantly different between patients above and below 55 years old after a median follow-up period of 18 months [19]. However, the mean age of patients in these studies was 66.9 and 63 years, respectively. These findings should thus be interpreted carefully because patients were relatively younger and the follow-up period was short. Other studies with older patients showed less benefit of percutaneous PFO closure compared to younger patients [4, 5]. However, these studies evaluated all causes of stroke recurrence, not only PFO-related cerebrovascular events. In addition, PFO morphology was unclear in these studies. Our study had an older sample (mean 75.2 ± 6.5 years old) and a longer follow-up period (mean 2.6 ± 1.8 years). Furthermore, only patients with high-risk PFO who performed percutaneous closure were assessed for recurrence of PFO-related cerebrovascular events. We suggest that routine percutaneous closure of elderly patients with PFO-related paradoxical embolism (not high-risk PFO) should not be recommended because of the unclear outcomes associated with this therapy, but high-risk PFO in older aged patients should be an indication to undergo percutaneous closure. Larger randomized trials are required to confirm our findings. However, it is necessary to determine the embolism source in both younger and older patients by TEE.

Previous study reported that percutaneous PFO closure under local anesthesia without balloon measurement or echocardiographic guidance was a safe and feasible procedure [20]. However, the study showed that total periprocedural complications rate was 2.5%, but contemporary device such as Amplatzer PFO Occluder (Abbott, Chicago, Illinois) was associated with complications < 1%. Amplatzer PFO Occluder is not clinically available in Japan. In this study, we performed percutaneous PFO closure using Amplatzer Cribriform device instead under general anesthesia with TEE guidance. To safely perform PFO closure without complications such as erosion and embolization, we re-assessed appropriate device size according to balloon sizing with TEE. As a result, there were no complications in our procedure.

Selection and duration of medication after PFO closure are also a controversial issue. There is no evidence of the superiority of either antiplatelet or anticoagulant therapy after PFO in the prevention of stroke recurrence [21]. In the elderly, however, hemorrhage is generally a more significant risk. For that reason, in our study population, approximately one-third of patients were not using antiplatelet and/or anticoagulant medication 6 months after PFO closure. As a result, no recurrence of cerebrovascular events occurred in our study. A previous trial also showed that despite 17% of patients being unmedicated 12 months after PFO closure, there was no stroke recurrence [7]. In our study, one patient showed cerebral hemorrhage, although no relation could be established between PFO closure and medication.

The increased risk of new-onset AF occurring usually within 45 days after percutaneous PFO closure was demonstrated in various studies [2, 22, 23]. However, previous studies showed that approximately three quarters of new-onset AF episodes do not progress to persistent [24], and stroke caused by AF related to PFO device closure is rare [25]. In our study, one patient suffered AF at one-month follow-up, but there was no recurrence of stroke on anticoagulant medication during the study's follow-up period.

Wahl et al. showed PFO closure reduces mortality compared to before/without closure [26]. In our study, all of the patients after PFO closure were survival. Although further studies with larger samples are required to confirm this point, PFO closure in elderly may be related to improve prognosis associated with PFO-related stroke.

We believe that older patients with high-risk PFO should undergo percutaneous closure to prevent recurrent cerebrovascular events without procedural complications. Patients with similar morphology should benefit from PFO closure, regardless of age.

### Study limitations

There are several limitations to this study. First, the number of patients was small, and this was a retrospective single-center study with no comparison of medical therapy alone without percutaneous PFO closure. Since it is sometimes difficult for older patients to perform TEE due to dysphagia and gas reflex, difficulties existed in detecting high-risk PFO and including a large number of patients in this study. Second, potentially AF was not completely excluded before percutaneous PFO closure procedure. However, no patients had symptoms of arrhythmia, all patients underwent cardiac monitoring until discharge, and none had any atrial high rate episodes. Moreover, stroke type was evaluated by MRI before procedure, and the neuroimaging pattern of AF was distinguished [27]. Third, our study population included older patients with relatively few comorbidities. Therefore, selection bias was present. Fourth, we used a single device (25 mm Amplatzer Cribriform). This was due to the fact that all PFOs in our study could be treated with this device. It is possible that Amplatzer PFO Occluder could lead to different results.

## Conclusion

Percutaneous transcatheter PFO closure for preventing recurrence of cerebrovascular events in elderly patients with high-risk PFO is a safe and effective treatment. It is also important to evaluate PFO morphology for elderly patients with paradoxical embolism. In addition, one-third patients could quit antithrombotic drug. These patients will avoid critical bleeding risk.
