# Correction to: Percutaneous transcatheter closure of high-risk patent foramen ovale in the elderly

**DOI:** 10.1007/s00380-019-01408-y

**Published:** 2019-04-15

**Authors:** Hiroya Takafuji, Shinobu Hosokawa, Riyo Ogura, Yoshikazu Hiasa

**Affiliations:** Department of Cardiology, Tokushima Red Cross Hospital, 103 Irinokuchi, Komatsushima-cho, Komatsushima, Tokushima 773-8502 Japan

## Correction to: Heart and Vessels 10.1007/s00380-019-01379-0

In the original publication of the article, the below sentence were garbled.

A list of errata is as follows:

1. In Abstract the sentence from “Between September 2012 and October 2018, 14 patients 60 years old with high-risk PFO underwent percutaneous closure to prevent recurrence of cerebrovascular events” is wrongly published, the correct sentence is as follows “Between September 2012 and October 2018, 14 patients ≥ 60 years old with high-risk PFO underwent percutaneous closure to prevent recurrence of cerebrovascular events”.
2. In Introduction the sentence from “However, most studies did not include elderly patients (age 60 years)” is wrongly published, the correct sentence is as follows “However, most studies did not include elderly patients (age ≥ 60 years)”.
3. In Methods under the section “Indication of percutaneous PFO closure and definition of high-risk PFO” the sentence from “If these TEE findings were confirmed, the brain–heart team consisting of an interventional cardiologist and neurologist discussed whether to perform percutaneous closure or medical follow-up, also taking into account each patientâ€™s frailty and activities of daily living performance” is wrongly published, the correct sentence is as follows “If these TEE findings were confirmed, the brain–heart team consisting of an interventional cardiologist and neurologist discussed whether to perform percutaneous closure or medical follow-up, also taking into account each patient’s frailty and activities of daily living performance”.
  The sentence starting from “Shunt bubble grade was classified according to the number of passing bubbles from the right to the left atrium: grade 0, none; grade 1, 1–5 bubbles; grade 2, 6–20 bubbles; grade 3, 20 bubbles” has been wrongly published, the correct sentence is as follows “Shunt bubble grade was classified according to the number of passing bubbles from the right to the left atrium: grade 0, none; grade 1, 1–5 bubbles; grade 2, 6–20 bubbles; grade 3,  ≥ 20 bubbles”.
  Also the sentence from “We defined high-risk PFO as follows: (1) long tunnel 10 mm and PFO separation height 2 mm” to “We defined high-risk PFO as follows: (1) long tunnel ≥ 10 mm and PFO separation height ≥ 2 mm”.
4. Methods
  Medications after percutaneous PFO closure, the sentence from “After percutaneous PFO closure, medications were prescribed according to the interventional cardiologist and neurologistâ€™s judgment.” Has been wrongly published, the correct sentence is as follows “After percutaneous PFO closure, medications were prescribed according to the interventional cardiologist and neurologist’s judgment”.
5. In Table 2, the second column is wrongly published as “Long tunnel â‰§ 0 mm (%)”, the correct line is “Long tunnel ≥ 10 mm (%)”. Also the column four is wrongly published as “Height â‰§ 2 mm (%)” the correct sentence is as follows “Height ≥ 2 mm (%)”.
6. In References some reference are published incorrectly, the correct reference is as follows,
  1. Mas JL, Derumeaux G, Guillon B, Massardier E, Hosseini H, Mechtouff L, Arquizan C, Béjot Y, Vuillier F, Detante O, Guidoux C, Canaple S, Vaduva C, Dequatre-Ponchelle N, Sibon I, Garnier P, Ferrier A, Timsit S, Robinet-Borgomano E, Sablot D, Lacour JC, Zuber M, Favrole P, Pinel JF, Apoil M, Reiner P, Lefebvre C, Guérin P, Piot C, Rossi R, Dubois-Randé JL, Eicher JC, Meneveau N, Lusson JR, Bertrand B, Schleich JM, Godart F, Thambo JB, Leborgne L, Michel P, Pierard L, Turc G, Barthelet M, Charles-Nelson A, Weimar C, Moulin T, Juliard JM, Chatellier G, CLOSE Investigators (2017) Patent foramen ovale closure or anticoagulation vs. antiplatelets after stroke. N Engl J Med 377:1011–1021.
  2. Søndergaard L, Kasner SE, Rhodes JF, Andersen G, Iversen HK, Nielsen-Kudsk JE, Settergren M, Sjöstrand C, Roine RO, Hildick-Smith D, Spence JD, Thomassen L, Gore REDUCE Clinical Study Investigators (2017) Patent foramen ovale closure or antiplatelet therapy for cryptogenic stroke. N Engl J Med 377:1033–1042.
  3. Hart RG, Diener HC, Coutts SB, Easton JD, Granger CB, O’Donnell MJ, Sacco RL, Connolly SJ, Cryptogenic Stroke/ESUS International Working Group (2014) Embolic strokes of undetermined source: the case for a new clinical construct. Lancet Neurol 13:429–438.
  4. Bayar N, Arslan Ş, Çağırcı G, Erkal Z, Üreyen ÇM, Çay S, Köklü E, Yüksel İÖ, Küçükseymen S (2015) Assessment of morphology of patent foramen ovale with transesophageal echocardiography in symptomatic and asymptomatic patients. J Stroke Cerebrovasc Dis 24:1282–1286.
  5. Spies C, Khandelwal A, Timmemanns I, Kavinsky CJ, Schräder R, Hijazi ZM (2008) Recurrent events following patent foramen ovale closure in patients above 55 years of age with presumed paradoxical embolism. Catheter Cardiovasc Interv 72:966–970.
  6. Wahl A, Jüni P, Mono ML, Kalesan B, Praz F, Geister L, Räber L, Nedeltchev K, Mattle HP, Windecker S, Meier B (2012) Long-term propensity score-matched comparison of percutaneous closure of patent foramen ovale with medical treatment after paradoxical embolism. Circulation 125:803–812.

The original article has been corrected.

